# Supporting public involvement in defining estimands: a practical tool accessibly explaining the five key attributes of an estimand

**DOI:** 10.1186/s13063-025-08941-4

**Published:** 2025-10-27

**Authors:** Suzie Cro, Eleanor Van Vogt, Nikki Totton, Ellen Lee, Jo C, Paul Hellyer, Manos Kumar, Yasmin Rahman, Ania Henley

**Affiliations:** 1 Imperial Clinical Trials Unit, School of Public Health, Imperial College London, London, UK; 2 Sheffield Centre for Health and Related Research (SCHARR), University of Sheffield, Sheffield, UK

**Keywords:** Clinical trial, Estimand, Patient and public involvement

## Abstract

**Background:**

An estimand is a precise description of the treatment effect a trial is aiming to find out. We previously identified that public partners (defined as patients and/or members of the public who are part of the research team) want to be involved in establishing estimands during trial planning. This involvement helps to ensure that trials address the questions that matter most to patients and the public. To initiate this, we co-developed a tool with public partners to help researchers explain the concept of an estimand in an accessible way. However, for public partners to be actively involved in defining estimands, the scientific terms used to describe the five attributes of an estimand must be further broken down. Accessible terms to describe estimand attributes would also be of benefit to researchers who are new to the estimand framework. Therefore, we aimed to co-develop with public partners an additional practical tool to clearly describe these five attributes and facilitate their understanding.

**Methods:**

An online consultation meeting followed by an in-person workshop was held with 5 public partners of mixed age, gender and ethnicities, from various regions of the UK. Public partner opinions were collected, and the newly proposed accessible terms to describe the attributes of an estimand were developed. Afterwards, the proposed accessible terms were presented to an independent wider patient and public involvement and engagement group with 15 public members at an online meeting. The accessible estimand attribute terms were refined and additional feedback sought via email.

**Results:**

A tool explaining the 5 attributes of an estimand, accessibly referred to as the 5 pillars of the research question, was created incorporating the public partner feedback.

**Conclusion:**

We provide a co-developed tool for researchers and public partners to use to facilitate the involvement of public partners in devising estimands. The tool explains the 5 attributes of an estimand using accessible terms proposed by public partners. It can be used in conjunction with the previously developed tool, which introduces what an estimand is and why it matters, to facilitate discussions with public partners on defining estimands during trial planning. The tools can also be used by other stakeholders including researchers unfamiliar with the estimand framework and those who find the scientific estimand attribute terms inaccessible.

**Supplementary Information:**

The online version contains supplementary material available at 10.1186/s13063-025-08941-4.

## Background

Estimands are used in the planning of clinical trials to ensure trials address the key questions of interest. An estimand is a precise description of the treatment effect a trial is aiming to find out, i.e. the exact research question investigated in a trial [1]. For a detailed primer on estimands with examples, see Kahan et al. [2]. By being clear about the estimands of interest in a trial, the trial can then be designed, conducted, and analysed using methods that enable the most important questions of interest to be addressed. Focussing on estimands provides a means to ensure trials provide relevant and meaningful results to all stakeholders, including patients and the public.

Additionally, when trial results are reported, estimands also help to ensure no misinterpretation of trial results [3]. Without the use of estimands it has been identified that most often it is unclear precisely what trials are investigating [4, 5]. This matters because asking different questions can lead to different answers on treatment benefit. For example, the effect of an intervention if all doses were received can be quite different from its effect when not all doses are received [2, 4, 6]. For all these aforementioned reasons, internationally adopted trial regulatory guidelines (ICH E9(R1)) call for trialists to include estimands during trial planning [1].

Public partners are defined as patients and/or members of the public who are part of the research team or advise the research team (not trial participants). In previous work, we explored public partner perspectives on discussing and defining estimands with public partners during trial planning [7]. We identified that public partners want to be involved in establishing estimands during clinical trial planning so that trials address what patients and the public want to know. To enable involvement of public partners in discussions in estimands, we co-developed a tool with public partners explaining the concept of an estimand and why estimands matter. This tool is freely available for researchers and public partners to use [7].

Whilst this tool can be used to start a conversation about what an estimand is with public partners, further estimand language, including the individual attributes of an estimand, is technical and less accessible. To completely specify an estimand, five attributes are required: (i) the population of interest, (ii) the treatment conditions being compared, (iii) the outcome/endpoint measure, (iv) the handling of intercurrent events, and (v) the statistical summary measure[1]. Intercurrent events are defined as post-baseline events that affect either the interpretation of or existence of trial outcomes. Therefore, to support public partners in devising estimands in trials, it is essential to further break down these scientific terms and clearly explain the five attributes of an estimand. To achieve this, we aimed to co-develop an additional practical tool with public partners that clearly describes these attributes, enabling public partners to actively participate in the definition of estimands. Accessible terms to describe estimand attributes would also be of benefit to researchers who are new to the estimand framework. In this article, we introduce and describe the co-development of this new tool, which incorporates more accessible language proposed by public partners.

## Methods

This study is reported following GRIPP2 guidelines [8]. An online consultation meeting was held in January 2023, followed by an in-person workshop in October 2023 with researchers (SC, EVV) and public partners (AH, JC, PH, MK, YR) from an established statistical trial methodology project, the HEALTHY STATS public involvement group. The group included five public partners aged between 20 and 70 years of mixed ethnicities and sex. Details on the remit and history of the HEALTHY STATS public involvement group have been described previously [7].

The objective of the first online meeting was to:

- Review the five attributes of the estimand and consider new accessible terms to use with public partners to describe these.

The Zoom platform was used for the online meeting, which lasted 2 hours. All discussions were audio-recorded. Each estimand attribute was presented to the public partner group. Public partners’ discussion points and feedback on the estimand language for each attribute were then collected from open-ended questions and Zoom polls to capture consensus. Following the online meeting, the accessible terms proposed in the meeting were written up by the lead researcher (SC).

The objectives of the second in-person workshop were to:

- Review the public partner proposed accessible terms to describe the attributes of the estimand from the previous online meeting.
- Finalise public partner proposed accessible terms to describe the attributes of the estimand.
- Co-develop a tool to explain the attributes of the estimand using the accessible terms.

Following the in-person workshop, the refined proposed accessible terms and a one-page tool to explain the attributes of an estimand were written up by the lead researcher.

The proposed new public partner accessible terms to describe the estimand attributes were then presented to an independent patient and public involvement and engagement (PPIE) group at The University of Sheffield (PPIE Methodology Group). This was to obtain further feedback from those that had not been involved in previous development at an online meeting (March 2024). The objective of this online meeting was to:

- Review the public partner accessible proposed terms to describe attributes of the estimand with an independent group public members and refine if indicated.

The Google platform was used for the online meeting. This meeting was one of the quarterly meetings that take place for the PPIE Methodology Group. Multiple projects were discussed at the meeting in addition to gaining feedback for this project. Discussion on the proposed accessible estimand attribute terms lasted 0.5 hour. The group included 15 members of the public with a mix of age and genders and researchers (including SC, NT, EL).

Following this meeting the public partner proposed accessible terms and tool were updated and finalised by the lead researcher. This was shared with the HEALTHY STATS group via email for approval and final feedback.

## Results

### Online consultation meeting

The language used by researchers to describe the five attributes of an estimand was presented to the HEALTHY STATS group (see first column of Table 1). It was immediately clear that the language needed to be made more accessible for public partners; terms alone were not adequate for public partners to understand each attribute. Each attribute was discussed in depth, and the initial more accessible terms devised by the public partners for each attribute are displayed in Table 1, column 3.

**Table 1:** Proposed accessible terms for estimand attributes for use with public partners

Attribute number	Estimand attribute (used by researchers)	Initially proposed accessible terms by public partners	Second draft proposed accessible terms by public partners	Final proposed accessible terms by public partners
1	The treatment conditions	What is the trial comparing	What is the trial testing	What is the trial comparing
2	The population	For who	What people/condition are we trying to help	What medical condition/people are we trying to help
3	The variable (or endpoint/outcome)	What difference is being measured	What is being measured	What is being measured
4	The handling of Intercurrent events	What is being done about expected/unexpected events that happen to patients in the trial	How are researchers handling unplanned participant related events, e.g. stopping prescribed medication early, taking other non-trial medications	What important events might happen during the trial and what should we investigate given they occur, e.g. stopping prescribed medication early, taking other non-trial medications
5	Population-level summary measure	What statistical measure are we using*	N/A	What statistic are researchers calculating, e.g. difference in proportion or average (typical) difference

^*^Uncertain whether this was necessary for public partners to know

To summarise, the first ‘treatment conditions’ attribute was felt to be confusing by public partners; to them, the ‘condition’ label suggested the underlying illness/medical condition, but this is not what this attribute refers to. This rather intends to capture the different interventions that are being compared in the trial. The ‘treatment’ term also indicated a cure to one individual, which may not always be the case. ‘What is the trial comparing’ was proposed as a better, more accessible alternative descriptor to eliminate doubt about what this attribute is referring to.

It was felt that the ‘population’ could simply be expressed as ‘For who’. The ‘outcome’ term by itself was not clear what this was referring to for public partners. They agreed it would be more understandable to use ‘what difference is being measured’. ‘Handling of Intercurrent events’ was the most unclear attribute to public partners. A preferred alternative was ‘What is being done about expected/unexpected events that happen to patients in the trial’; however the group agreed this was not the best alternative and decided they would like to revisit this at a subsequent meeting. Instead of ‘Population-level summary measure’, the group considered it more informative to use ‘What statistical measure are we using’. However, there was also debate amongst the group as to whether this fifth attribute was useful for public partners to have a say on, or be involved in discussions on. Therefore, there was uncertainty as to whether an accessible lay term was needed for this attribute. It was agreed the initial accessible term proposals from this first meeting would be revisited at a second meeting.

### In-person workshop

At the following in-person workshop, the proposed accessible terms from the first online meeting were each discussed further. The second resulting more accessible terms refined during the in-person workshop are displayed in Table 1, column 4. There was a group consensus to refer to the attributes of the estimand as ‘the pillars of the research question’. It was agreed that ‘what is the trial testing’; would be preferable to ‘what is the trial comparing’ for the first attribute referred to by researchers as ‘treatment conditions’. For the second ‘population’ attribute, the group wanted to make this more understandable and to humanise it. It was discussed how researchers could intend the population to capture what condition (e.g. eczema) and, where relevant, key demographics (e.g. age) the study intends to target the treatment effect for. Both elements were important to capture, so the more accessible term was updated from ‘For who’ to ‘What people/condition are we trying to help’ so that it is clear what the population can include. For the ‘outcome’ attribute, upon reflection from the group, it was not clear what ‘difference’ this is meaning, and it was felt ‘what is being measured’ would be adequate.

For ‘Handling of intercurrent events’, there was agreement this was still the trickiest attribute to understand and describe. Public partners understood that this could be thought about as what the statisticians/researchers do when something does not go to plan in the trial, i.e. when unplanned events occur or unexpected events. ‘Random events’ was suggested as an alternative way to describe intercurrent events, but was then dismissed as it could not be considered that such events are always random. ‘Unwanted events’ was considered too negative. The group settled on describing this attribute as ‘How are researchers handling unplanned participant-related events.’ Public partners felt it was important that researchers give examples of unplanned participant-related events alongside this description to aid understanding.

For the ‘Population-level summary measure’ attribute, the group decided it was not helpful for patients/public to consider this in trial planning. They considered this an attribute they would be happy for researchers to decide what was most applicable, so an accessible term would not be indicated. There were therefore four pillars of the research question deemed relevant to public partners and proposed with accessible lay terms at the end of the in-person workshop.

During this workshop, it was decided it would be useful to add a third page to the previously co-developed estimand explainer tool [7] to explain the four important pillars of the research question using the accessible lay terms. This is to facilitate public partners having a say on these four required elements in practice in trial planning. This third page was discussed and an initial draft formed on large A6 sheet of paper with a sketch of the proposed page. The use of graphics was suggested to make it appealing to review.

### Online wider PPI group meeting

The estimand attributes used by researchers and the more accessible terms used to describe these (Table 1) were presented to an independent group of public members. For the first attribute, ‘What is the trial testing’, individuals in this group did not like the term ‘testing’, they preferred the original ‘comparing’ term (see Table 1, column 3); the use of the word ‘testing’ made one individual think of human guineapigs being tested in a trial and they were consequently not keen on this term. The alternative of ‘evaluating’ was suggested, but then dismissed as considered ‘too professional’. So, the initial ‘What is the trial comparing’ (from meeting 1) was reverted to as considered a more positive term. For the second attribute, ‘What people/condition are we trying to help’ discussions revealed adding ‘medical’ prior to condition, would make this more contextually relevant and capture how referring to a medical condition that the research is aiming to address.

For ‘How are researchers handling unplanned participant-related events, e.g. stopping prescribed medication early, taking other non-trial medications’—public members questioned how such events might be planned as well as unplanned; for example, a doctor might plan to reduce a participants medication part way through the trial given their early experience in the trial. Public partners agreed this was the trickiest attribute to understand and indicated an update to this descriptor was required. In line with the HEALTHY STATS group, they also suggested it was useful to include examples alongside the description. Following the feedback, the accessible term was updated to, ‘What important events might happen during the trial and what should we investigate given they occur e.g. stopping prescribed medication early, taking other non-trial medications ‘

In contrast to the HEALHY STATS group, the PPIE Methodology Group felt the accessible terms should cover all five attributes of the estimand. Although they similarly agreed public partners might not be interested in defining the summary measure attribute, they felt it was important to have access to a non-technical translation of this attribute in case it comes up in conversations with researchers that public partners are also present for. It was agreed to include an accessible definition for this fifth attribute in the tool with examples as, ‘What statistic are researchers calculating e.g. difference in proportion or average (typical) difference’.

**Fig. 1:**
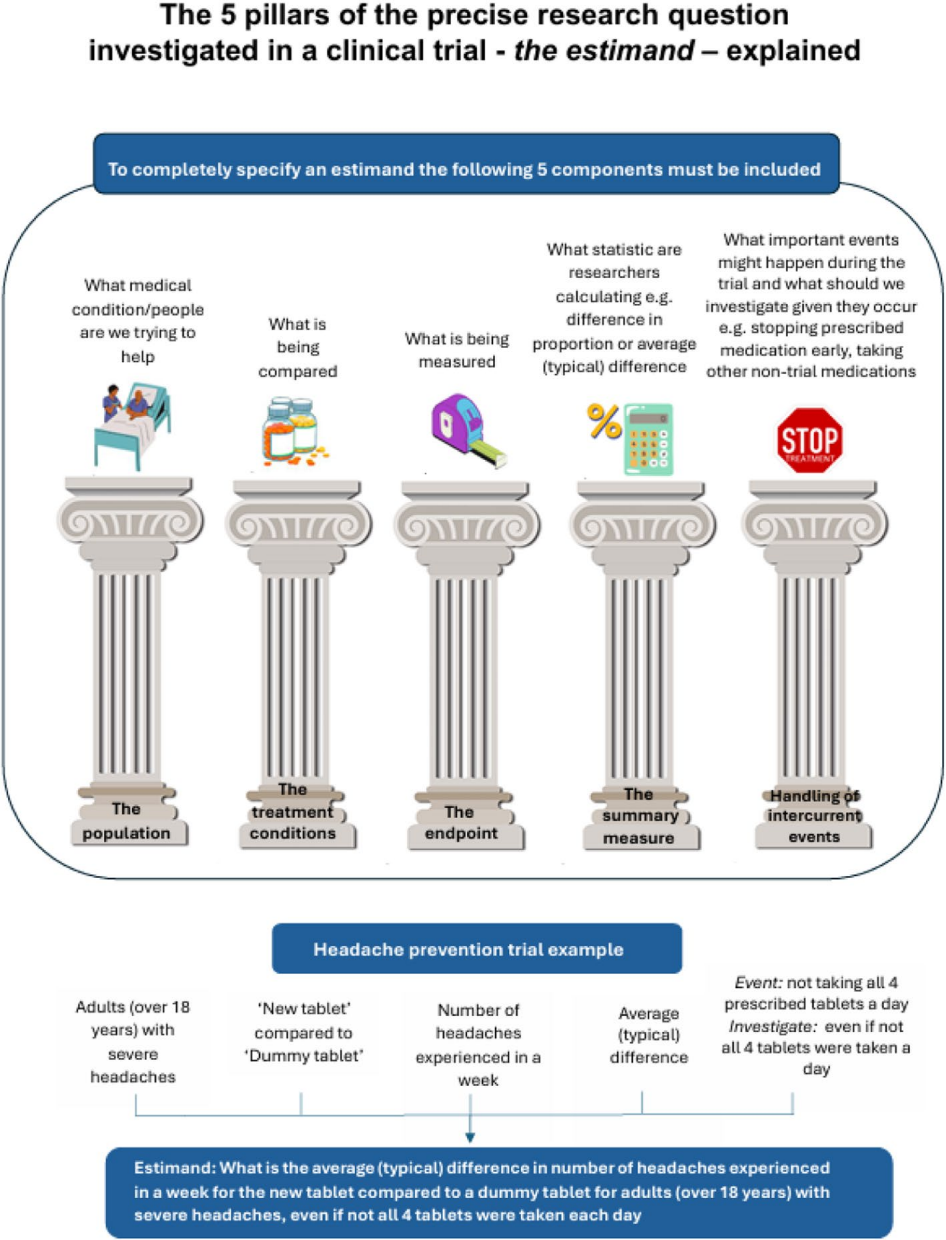
The 5 pillars of the precise research question investigated in a clinical—*the estimand*—explained

Following this third meeting, the agreed accessible lay terms were written up as a one-page tool with graphics refined to aid understanding—see Fig. 1, Table 1 (column 5) and Supplementary file 1 for a downloadable version. This was shared with HEALTHY STATS group via email for approval and any final feedback, with explanation of why the 5th attribute was added in. No objections or further feedback were obtained.

## Discussion

### Main findings

A practical tool was co-developed with the HEALTHY STATS group and the University of Sheffield PPIE Methodology group to explain the five attributes of an estimand using accessible lay terms. This tool, along with the previously developed one introducing what an estimand is and why it matters [7] can be used to facilitate discussions with public partners on defining estimands during trial planning.

Use of these tools is not restricted to public partners. They can also be used by other trial stakeholders, including clinical and non-clinical trial team members who are new to estimands to help understand the concept of an estimand, and the five attributes of an estimand. The tools can also be used to aid multi-disciplinary group discussion by using accessible terms. We have received feedback that these tools are also helpful beyond public partners to help explain the more complex and unfamiliar estimand language. Whilst some members of the public have told us they do not feel public partners necessarily need to contribute to deciding the 5th population level summary measure attribute in practice, having a translation of this attribute on the tool makes the tool widely useable. Further, it enables public partners to follow and understand wider conversations on this attribute if it is raised during meetings they are present at.

### Research in context

International trial regulatory guidelines (ICH E9(R1)) that are now adopted worldwide call for trialists to include estimands during trial planning [1]. Public partners have previously indicated they want to have a say on estimands to ensure trials address what is of interest to them [7]. The provided tools, co-developed with public partners, aim to enable this. It has been highlighted how multi-disciplinary collaboration is needed to implement the ICH E9(R1) framework and devise estimands [9]. Whilst guidance has been provided for researchers, this tool opens the door to including public partners in this multi-disciplinary effort to ensure trials address the needs of all stakeholders.

### Strengths and limitations

Public partner perspectives were essential to generate the new accessible estimand terms, which enable the estimand attributes to be accessible to other public partners. The fact that we went to the HEALTHY STATS group and a second independent larger group to review the terms is a strength of this study. Both groups included a mix of ages, genders and ethnicities from different parts of the UK. Online and in-person meetings worked similarly well to collect suggestions and feedback on accessible terms. In total, 20 public partners contributed to these new accessible terms. We acknowledge that this is limited, but similar feedback and discussion points were raised by both groups. There were also some differences, for example, on whether a definition was indicated for the ‘population level summary measure’ attribute. However, the differences led to careful consideration of this attribute and, ultimately, a more comprehensive and usable tool.

### Future research

The next steps are to use these tools with different groups of public partners in a range of applied trial design contexts to evaluate performance and implementation. We welcome readers who use the tool to contact the corresponding author of this article (SC) to provide feedback on its implementation.

As noted by our public partners discussions, how to handle intercurrent events is the most complex attribute for public partners to have a say on. We have proposed new terms to discuss this attribute. After identifying relevant events, there are different strategies that can be used to handle such events. These similarly have technical terms: treatment policy, hypothetical, principal stratification, composite and whilst on treatment [1]. Understanding these strategies was beyond the scope of this project, which explored the five given attributes of an estimand. The best way to devise intercurrent event strategies with public partners in a trial design context needs to be established and is now the focus of further work.

## Conclusions

To facilitate and therefore encourage the involvement of public partners in defining estimands in trial planning, we co-developed a tool explaining the five attributes of an estimand, accessibly referred to as ‘pillars of the research question’, which is available for researchers and public partners to use. This tool can also be used by other researchers new to estimands, providing more accessible terms for describing estimand attributes.

## Supplementary Information

Supplementary Material 1.

Supplementary Material 2.

## Data Availability

All data and materials are included in this publication.

## Abbreviations

HEALTHY STATS: Public involvement group set up to support an NIHR advanced fellowship (NIHR300593) to develop accessible statistical methods to determine treatments effects that matter to patients and prescribing physicians in randomisedcontrolled trials.
ICH E9(R1): International council for harmonisation of technical requirements for pharmaceuticals for human use addendum on estimands and sensitivity analysis in clinical trials to the guideline on statistical principles for clinical trials.
PPIE: Patient and public involvement and engagement
