# Supplementary material for: Supporting public involvement in defining estimands: a practical tool accessibly explaining the five key attributes of an estimand
Source: Trials. 2025 Oct 27;26:440. doi: 10.1186/s13063-025-08941-4 (PMC12560361; doi:10.1186/s13063-025-08941-4)
Supplement: Supplementary file 1 — Supplementary Material 1. [file 13063_2025_8941_MOESM1_ESM.pdf]

The 5 pillars of the precise research question investigated in a clinical trial - *the estimand* – explained

To completely specify an estimand the following 5 components must be included

What medical condition/people are we trying to help

What is being compared

What is being measured

What statistic are researchers calculating e.g. difference in proportion or average (typical) difference

What important events might happen during the trial and what should we investigate given they occur e.g. stopping prescribed medication early, taking other non-trial medications

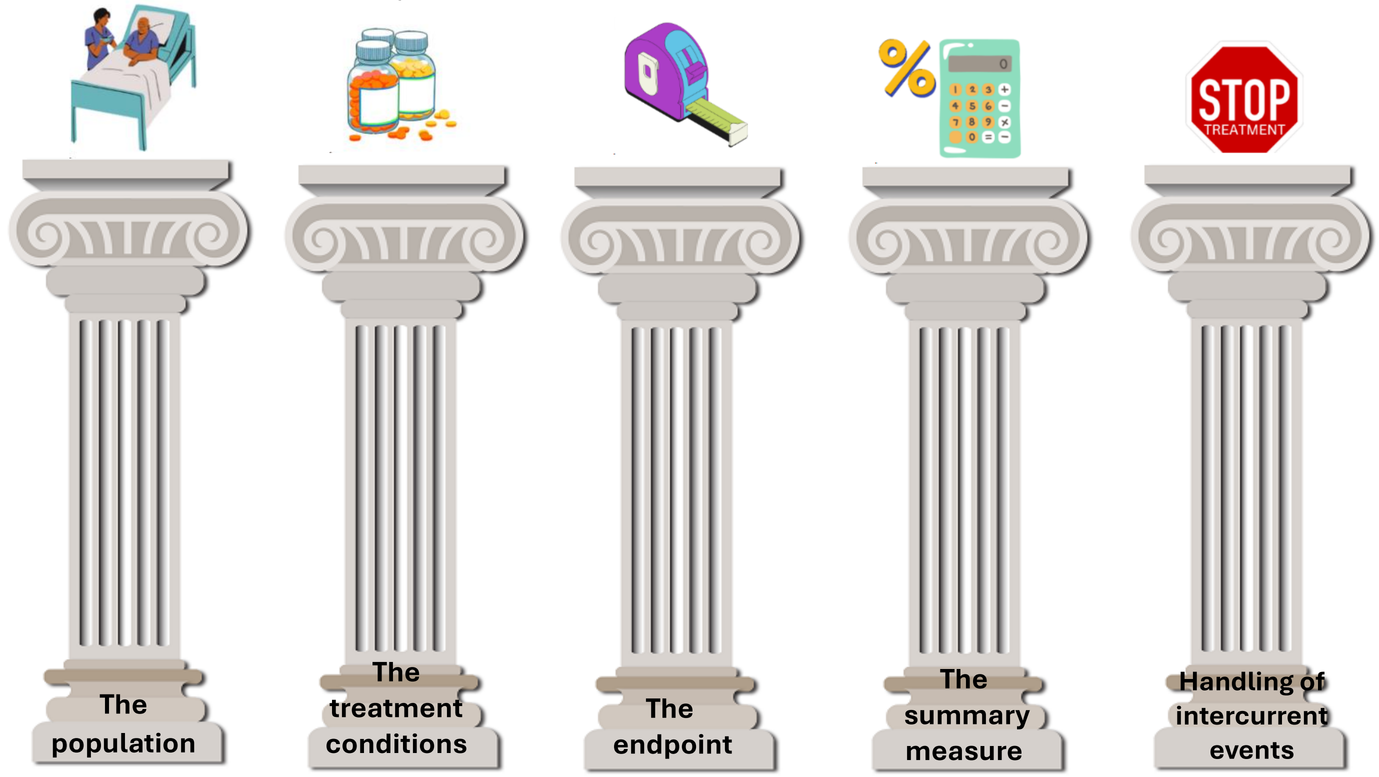

Headache prevention trial example

The population	Adults (over 18 years) with severe headaches
The treatment conditions	‘New tablet’ compared to ‘Dummy tablet’
The endpoint	Number of headaches experienced in a week
The summary measure	Average (typical) difference
Handling of intercurrent events	*Event:* not taking all 4 prescribed tablets a day *Investigate:* even if not all 4 tablets were taken a day

Estimand: What is the average (typical) difference in number of headaches experienced in a week for the new tablet compared to a dummy tablet for adults (over 18 years) with severe headaches, even if not all 4 tablets were taken each day
