# Supplementary material for: Supporting public involvement in defining estimands: a practical tool accessibly explaining the five key attributes of an estimand
Source: Trials. 2025 Oct 27;26:440. doi: 10.1186/s13063-025-08941-4 (PMC12560361; doi:10.1186/s13063-025-08941-4)
Supplement: Supplementary file 2 — Supplementary Material 2. [file 13063_2025_8941_MOESM2_ESM.docx]

GRIPP2 short form

| Section and topic | Item | Reported on page No |
| --- | --- | --- |
| 1: Aim | Report the aim of PPI in the study | 4 |
| 2: Methods | Provide a clear description of the methods used for PPI in the study | 4-6 |
| 3: Study results | Outcomes—Report the results of PPI in the study, including both positive and negative outcomes | 6-11 including Table 1, Figure 1 and supplementary file 1 |
| 4: Discussion and conclusions | Outcomes—Comment on the extent to which PPI influenced the study overall. Describe positive and negative effects | 12-15 |
| 5: Reflections/critical perspective | Comment critically on the study, reflecting on the things that went well and those that did not, so others can learn from this experience | 13-14 |

PPI=patient and public involvement
